# Report of Two Cases of Ovariotomy Successfully Performed

**Published:** 1882-03

**Authors:** Matthew D. Mann

**Affiliations:** Professor of Obstetrics and Gynecology, University of Buffalo


### Report of two Cases of Ovariotomy Successfully Performed.

By Matthew D. Mann, A. M., M. D., Professor of Obstetrics and Gynecology, University of Buffalo.

*Case I.*—Miss L. B., æt. 27, single. Has always menstruated regularly. Seven years ago she had a large abdominal swelling which lasted some months; was considered to be dropsical in its character, but disappeared under the use of a quack remedy. Since that time has always been in good health. First noticed present abdominal enlargement 18 months ago. She has never suffered under any pain from it, only inconvenience from its size. During the last six months it has grown very rapidly. On examination the patient presented the appearance of a woman in the last stages of pregnancy. The abdomen was distended by a large fluid tumor, which presented the usual characteristics of an ovarian tumor. The walls were very thin and fluctuation very distinct. The uterus was pushed upwards and forwards by the tumor and was not enlarged.

The diagnosis was first made by Prof. Thomas F. Rochester, who referred the case to me for operation.

Before operating, in order to remove, if possible, all doubts as to the nature of the swelling, a small quantity of fluid was drawn off by an aspirator. This fluid coagulated under heat and contained the cells usually found in ovarian fluids.

The patient having been prepared by a thorough clearing out of the bowels, the operation was performed January 4, 1882, before the medical class of the University, with the aid of Professors Rochester and Cary and the house staff of the hospital. Professors Moore, Miner, and a number of other gentlemen were present.

The carbolic spray was dispensed with, but in other respects the antiseptic method was carried out. The patient being etherized, an incision was made through the abdominal walls about half way between the pubes and navel. The incision was made with a scalpel and grooved director. When the sack was reached, an exploration of the abdominal cavity with a sound, passed between the tumor and the walls, showed no adhesion in the anterior surface. The tumor was tapped, but only a small quantity of fluid would pass through the trocar. The patient was therefore turned on her side. The cyst wall was seized with forceps, and the portion of it, around the hole made by the trocar, drawn into the abdominal wound. By enlarging the opening a little a considerable quantity of fluid flowed out, but it was found necessary to enlarge the abdominal wound, and the opening into the cyst, enough to allow of the admission of the hand. It was then discovered that the cyst was multilocular, and that the trocar had been stopped by some small pedunculated cysts getting into the end. These were held back, the patient turned again on her side, and the cyst soon emptied. On attempting to draw the sack out of the peritoneal cavity, it was found to be strongly adherent to a portion of the small intestines, to the omentum and to the sides of the pelvis. At this point two methods of procedure presented themselves for adoption. Either to attempt to enucleate the cyst, or to cut off all the unattached portion and to stitch the pocket that was left to the edges of the abdominal wound, insert a drainage tube into it, and treat it as an open wound. The former method was decided upon. After cutting into the band connecting the intestine to the cyst-wall, it was peeled off and the process of peeling carried deeper and deeper until the bottom of the attachment was reached. The sides and remaining portions were then detached by the fingers, in the same way, all except one very large and firm adhesion to the uterus. This was transfixed and tied with a strong silk ligature. The attachment to the omentum was tied with carbolized cat-gut and cut.

After the cyst was removed, the abdominal cavity was cleaned with the greatest care; all blood clots were removed, and bleeding points searched for and tied. A glass drainage tube was then passed down into Douglas' cul-de-sac and the wound closed with silver wire.

The patient stood the operation remarkably well, considering its severity and the length of time (one hour and a half) which it lasted.

She recovered from the ether quickly and well, and eight hours afterward was found to have a pulse of 102, temp. 102½°. The next day the pulse reached 130 and the temperature 103¼°, but both soon fell, and after the second day the temperature but once went above 101½° and the pulse above 120, and most of the time both were considerably lower. It was not, however, until the tenth day that the evening exacerbations ceased, and the pulse and temperature became normal.

The diet for the first thirty-six hours consisted entirely of small quantities of brandy and ice. The urine was drawn, and pain and restlessness quieted with occasional small doses of opium or chloral.

The tube was removed on the evening of the fourth day, all discharge through it having ceased. The lower part of the sinus left by its removal closed at once, but when the patient was last seen, a small opening about two inches deep, just large enough to admit a probe, still remained. The rest of the wound closed by primary union, and the stitches were removed at the end of a week. The patient made steady progress. Bowels moved on the ninth day by enema. On the fifteenth day there was a slight diarrhœa; on the 18th day she sat up in bed, and a few days afterward sat in the chair. At the end of four weeks she left the hospital perfectly recovered.

*Case II.*—Mrs. R. C. S., æt. 35, married two years, was delivered of a seven-month's fœtus last September. She first noticed the tumor about a year ago, about the time that she became pregnant. She enlarged very rapidly, and in July had some abdominal pain for which a physician was called. He diagnosed the case as one of dropsy. Two weeks later Dr. S. D. Freeman recognized the presence of an ovarian cyst and of pubic pregnancy. Later in the month she felt life, and in September the waters broke. Three weeks afterward labor came on; it was rather long, but normal. After the child was born she diminished only two inches around the abdomen; present measurement 46 inches.

On examination a fluctuating cyst could be felt, and there were the usual signs of ovarian tumor. On inspection the abdomen presented a somewhat peculiar appearance. It was rather flattened on top and spread out on the sides, presenting very much the appearance found in ascites. A portion of the fluid was drawn off by the aspirator and found to contain the same elements and to have the same reaction as that of the first case.

The operation was performed February 1st at the General Hospital, with the aid of Drs. Cary and Mynter and the house staff, and in the presence of Profs. Moore and Miner, Drs. Gay, Wyckoff, and a number of other gentlemen. An incision was made in the usual manner through the abdominal walls. A few adhesions were found near the liver, between the cyst and abdominal wall. The fluid flowed freely through the trocar, and after it was emptied, there was found a mass of small cysts growing into the main cyst from its walls. These were too large to bring through the wound, and were therefore broken up by the hand introduced into the cyst. After this the sack was easily pulled out, the adhesion broken and the pedicle exposed. This was found to be small, and was transfixed, tied with silk, and dropped. On examination the right ovary was found to be enlarged and to contain a cyst the size of a horsechesnut. It was therefore thought best to remove it. The pedicle was tied with cat-gut and an incision made so as to leave a certain part of the ovarian structure, a part which seemed perfectly healthy. The abdomen was carefully cleansed, clots removed, and the wound closed with six silver sutures.

The patient stood the operation remarkably well. Soon after it was over the pulse was found to be 84, and it never rose above 90. On the second day the temperature went to 100½. The next day it fell to 100, and never went above that point again. Since the sixth day it has been normal. Three sutures were removed on the sixth day, and the remainder on the eighth. The wound was perfectly united. The after-treatment was the same as that carried out in the other case. The patient is now sitting up (twelfth day) and steadily gaining in strength. Good appetite, regular bowels, and normal pulse and temperature. She still complains of a little pain over the right ovary.

*Remarks.* About the second case there is little to be said. The only difficulty was the diagnosis, which was slightly obscured by the appearance and shape of the abdomen. This peculiarity was accounted for by the fact that the abdomen had been very much stretched by the presence of the fœtus, and that being removed the tumor was not large enough to fill it. Hence the peculiar flattening and bulging at the sides.

As to the propriety of removing the other ovary, it may be said that the cyst which had developed was probably an enlarged Graafian follicle, and such tumors do not generally reach any great size. Still it was better to remove it than to run the risk of having it develop, especially as its removal did not to any great extent increase the risk of the operation.

The method adopted for the removal of the cyst in the first case is certainly the best. This plan was first brought to the notice of the profession by Dr. Miner, and is declared by Thomas to be "one of the most valuable of all the contributions to ovariotomy which have emanated from this country." The alternative leaves a large portion of the cyst to be treated as an open wound. This closes very slowly and leaves the patient constantly exposed to the danger of septic absorption. In one case where I employed it the patient made a good recovery from the operation, but died some months later from septicæmia, due to a failure to keep the cyst well cleansed. Had the tumor been enucleated, I have no doubt that she would have remained well.

The use of the drainage tube was indicated from the very extensive oozing surface, which was left by the enucleation of the tumor. During the first two days a considerable quantity of bloody serum flowed away. To Drs. Fredericks and Pryor, of the General Hospital, my thanks are due for the skillful way in which they carried out the after-treatment.

